# Are Worry and Rumination Specific Pathways Linking Neuroticism and Symptoms of Anxiety and Depression in Patients with Generalized Anxiety Disorder, Major Depressive Disorder and Mixed Anxiety-Depressive Disorder?

**DOI:** 10.1371/journal.pone.0156169

**Published:** 2016-05-31

**Authors:** Hipólito Merino, Carmen Senra, Fátima Ferreiro

**Affiliations:** Department of Clinical Psychology and Psychobiology, University of Santiago de Compostela, Santiago de Compostela, A Coruña, Spain; Bellvitge Biomedical Research Institute-IDIBELL, SPAIN

## Abstract

This study examines the relationships between neuroticism (higher-order vulnerability factor), the cognitive styles of worry, brooding and reflection (second-order vulnerability factors) and symptoms of anxiety and depression in three groups of patients: patients with Generalized Anxiety Disorder (GAD), with Major Depressive Disorder (MDD) and with Mixed Anxiety-Depressive Disorder (MADD). One hundred and thirty four patients completed a battery of questionnaires including measures of neuroticism, worry, rumination (brooding and reflection), anxiety and depression. Multiple mediation analyses indicate that worry may act as a mediating mechanism linking neuroticism and anxiety symptoms in the three diagnostic groups, whereas brooding-rumination may play a mediating role between neuroticism and depressive symptoms in patients with MDD and MADD and, with less certainty, in patients with GAD. Overall, our findings suggest that neuroticism may increase the risk of anxious and depressive symptoms via specific links involving either worry or brooding, respectively, and that both worry and brooding may operate in the three groups examined, irrespectively of whether anxiety or depression are the main emotions or whether they coexist without any clear predominance; consequently, we hypothesize the existence of "specific transdiagnostic" mechanisms.

## Introduction

Generalized Anxiety Disorder (GAD) and Major Depressive Disorder (MDD) are two of the most common mental health disorders and are the most closely associated of all emotional disorders [1,2]. One of the proposed explanations for the high rate of comorbidity is derived from models of hierarchical structure [3]. Such models propose the existence of higher-order vulnerability factors (e.g. personality traits) and other relatively specific second-order factors that mediate between the former predispositional factors and different anxious and depressed manifestations. However, it is not clear whether second-order factors support a transdiagnostic focus, which suggests the involvement of common psychopathological processes in the genesis and maintenance of anxiety and depression [4], or whether second-order factors display specificity so that different vulnerability pathways result in either anxiety or depression, respectively [5]. Although great efforts have been made to clarify these questions, determining and differentiating between elements that are common to both disorders and those that are specific to each of them remains a challenge [6].

Among the higher-order vulnerability factors proposed by the hierarchical models, neuroticism has received an important empirical support as a predispositional factor for both anxiety and depression [7,8]. However, the explanatory function of neuroticism is reduced by its poor value as a marker of vulnerability [9]. Identification of second-order mechanisms via which neuroticism may lead to anxious and/or depressive outcomes would thus represent a step forward in the study of emotional disorders [10].

It is known that individuals who score high on neuroticism tend to experience negative emotional states that make them prone to intrusive and recurrent thoughts in order to confront the emotional discomfort [11]. In turn, these repetitive negative thoughts increase the vulnerability to suffering emotional disorders [12]. Worry and rumination are two types of repetitive thought processes that are widely associated with anxiety and depression. Worry, a defining trait in generalized anxiety disorder (GAD) [13], implies a chain of repetitive and uncontrollable thoughts aimed at preparing for and preventing possible future threats [14]. Rumination, characteristically associated with depression, represents a passive repetitive response to a depressive or dysphoric state related to past failures or errors [15]. The main difference between these constructs is that worry involves the future, implies a reaction to fear and may be motivated by an attempt to plan or anticipate uncertain situations [16]. By contrast, rumination is mainly directed to the past, involves loss or discrepancies between the current state and a desired state and is motivated by a wish to understand the emotional discomfort suffered [16]. The identification of two components of rumination–brooding and reflection–was an important advance in the conceptualization of this construct [17]. Brooding describes “a passive comparison of one’s current situation with some unachieved standard”, whereas reflection describes a “purposeful turning inward to engage in cognitive problem solving to alleviate one´s depressive symptoms” ([17], p. 256). Several studies have reported that brooding is associated both cross-sectionally and longitudinally with depressive symptoms [18]. However, evidence for the relation between reflection and depressive symptoms is less consistent. Thus, although some authors maintain that reflection is positively associated with depression [19], these findings have not been replicated in other studies [18,20].

Despite the above-mentioned differences between worry and rumination, these cognitive processes share many similarities as both are characterized by intrusive, repetitive, prolonged and uncontrollable thoughts about future or past experiences, with similar consequences at emotional, cognitive and interpersonal levels [21]. Several authors have reported that worry and rumination are related but statistically distinguishable constructs [22]; however, others have not found any significant differences between these processes [23]. It is also not clear whether worry and rumination have unique features that are differentially linked to anxiety and depression [24]. Traditionally, worry has been associated with anxiety [25] and rumination with depression [26]. However, new data suggest that worry is also associated with depression and rumination with anxiety [22,27]. Obtaining empirical evidence for the similarities and differences between these constructs remains an important research challenge, as does establishing how they are related to symptoms of anxiety and depression.

The present study examined the relationships between the general higher-order vulnerability factor of neuroticism, the second-order vulnerability factors of worry, brooding and reflection, and symptoms of anxiety and depression in three groups of patients, with the aim of testing the transdiagnostic and content specificity hypotheses. Patients diagnosed with a pure anxiety disorder (Generalized Anxiety Disorder, GAD), pure depressive disorder (Major Depressive Disorder, MDD) and Mixed Anxiety-Depressive Disorder (MADD) were selected for the study. According to some authors [28], the inclusion of patients diagnosed with “pure” disorders enables precise identification of the association between cognitive mechanisms and symptoms. The inclusion of the mixed group enables comparison of the nature of the relationship when the symptoms are comorbid, without clear predominance of one or other of these emotions. Specifically, the main aim of the present study was to explore the mediating effects of worry and rumination on the relationships between neuroticism and symptoms of anxiety and depression in patients diagnosed with pure disorders (GAD or MDD) and with Mixed Anxiety-Depressive Disorder (MADD). As rumination is a multidimensional construct, the differential role of its components (brooding and reflection) was analyzed.

## Materials and Methods

### Participants and procedure

A total of 134 patients (72% female) were recruited on an outpatient basis from the Psychiatric Services at the University Clinical Hospital of Santiago de Compostela (A Coruña, Spain), between January and August 2014. Those patients who met the criteria for diagnosis of Major Depressive Disorder, Generalized Anxiety Disorder or Mixed Anxiety-Depressive Disorder were chosen. Patients with primary diagnoses other than MDD, GAD or MADD and patients with any comorbid disorder were excluded from the investigation to control the coherence of the samples. A total of 45 patients met the diagnosis for GAD, 49 patients met the diagnosis for MDD and 40 patients met the diagnosis for MADD.

The mean age of the sample was 40.24 years (*SD* = 11.25, range 19–69). Forty-four per cent were married, 40.3% were single, 13.5% were separated or divorced and 2.2% were widowed. In terms of educational level, the distribution was as follows: 31.3% had a low level of education, 33.6% had an average level of education and 35.1% had a high level of education.

Patients were consecutively admitted to the study if they met the inclusion criteria. The diagnoses were made by following the Structured Clinical Interview for DSM-IV-TR axis 1 diagnoses [29], which was administered by trained clinical psychologists familiar with the DSM-IV-TR classification and diagnostic criteria [30].

Before receiving any form of treatment, 141 patients completed the battery of self-report measures under the supervision of a member of staff. Seven of the patients were excluded from the study because they did not complete all of the questionnaires.

Approval for the study was granted by the Bioethics Committee at the University of Santiago de Compostela. Written informed consent was obtained from the patients after they were provided with a full description of the study.

### Materials

#### Eysenck Personality Questionnaire-Neuroticism [EPQ-N]

The EPQ-N is a self-report that consists of 12 dichotomous items designed to measure the personality dimension of neuroticism [31]. The Spanish version, which has proved to be reliable and valid [32], was used. The reliability, calculated by the Kuder-Richardson formula [33], was .78.

#### Penn State Worry Questionnaire [PSWQ]

The PSWQ is a 16-item self-report for assessing the tendency to engage in excessive, generalized and uncontrollable worry [34]. The Spanish version, for which satisfactory validity and reliability have been reported [35], was used. Cronbach’s alpha was .95.

#### Ruminative Response Scale [RRS]

The RRS is a 22-item self-report for assessing ruminative coping responses to depressed mood [36]. Given that it was found that 12 items from the RRS overlapped with depressive symptoms [17], the resulting 10-item version was used. An exploratory factor analysis on these remaining 10 items supported a two-factor model: Brooding (five items) and Reflection (five items) [17]. The Spanish translation of the RRS, which has been found to show adequate psychometric properties [37], was used. Cronbach’s alpha scores for brooding and reflection were .79 and .76, respectively.

#### State-Trait Anxiety Inventory, Trait version [STAI-T]

The STAI-T is a 20-item self-report for assessing anxiety as a trait [38]. The Spanish version, which has demonstrated satisfactory psychometric properties [39], was used. Cronbach’s alpha was .89.

#### Beck Depression Inventory-II [BDI-II]

The BDI-II is a 21-item self-report for measuring the severity of current depressive symptomatology [40]. The Spanish version, which has been shown to exhibit high psychometric quality [41], was used. Cronbach’s alpha was .89.

## Results

### Preliminary analyses

The data were analyzed to determine whether they met all of the statistical assumptions [42]. Assumptions of linearity, normality and homogeneity of variance were fulfilled for all measures.

A univariate ANOVA was conducted considering the diagnostic group as the fixed factor and the scores for all measures studied as the dependent variables. The descriptive statistics and the post hoc comparisons between means are included in Table 1. The mean scores (neuroticism, cognitive styles and anxiety and depressive symptoms) are representative of scores obtained in other clinical samples [43]. Specifically, the three groups had high and similar scores for neuroticism. Not surprisingly, the scores for worry were significantly higher in patients with GAD than in patients with MDD and scores for brooding were significantly higher in patients with MDD than in the other groups. By contrast, the scores for reflection did not differ significantly between groups, although they were slightly higher in the patients with MDD.

**Table 1:** Means, Standard Deviations and Comparison of the Study Variables by Diagnostic Group. GAD, Generalized Anxiety Disorder; MDD, Major Depressive Disorder; MADD, Mixed Anxiety-Depressive Disorder.

Variables	*M* (*SD*)			Post-hoc Comparisons	
	GAD (*n* = 45)	MDD (*n* = 49)	MADD (*n* = 40)	*F*	Significant differences
Neuroticism	8.7 (2.7)	8.6 (2.5)	9.2 (2.1)	0.66	
Worry	62.1 (13.0)	54.7 (14.5)	55.8 (13.8)	3.76*	GAD > MDD
Brooding	12.0 (5.2)	15.1 (4.3)	12.0 (3.2)	7.47**	MDD > MADD, GAD
Reflection	10.3 (3.8)	11.8 (2.6)	10.3 (2.5)	3.60	
Anxiety symptoms	41.1 (11.1)	35.6 (9.4)	39.3 (10.1)	3.53*	GAD > MDD
Depressive symptoms	22.2 (10.2)	32.9 (11.7)	26.5 (9.3)	12.25***	MDD > MADD, GAD

*p ≤ .05.

**p ≤ .01.

***p ≤ .001.

The Pearson’s correlations between the different variables in each diagnostic group are shown in Table 2. Neuroticism was significantly correlated with the cognitive styles worry and brooding (but not reflection) and with symptoms of anxiety and depression in all groups of patients. The cognitive styles worry and brooding, unlike reflection, were significantly associated with both anxiety and depression in all the cases, except worry in the MDD group which was significantly associated with anxiety symptoms only. The specificity of worry and rumination for the anxiety and depressive symptoms was tested with Steiger´s *z* tests. The results of these tests showed that worry was more strongly associated with anxiety symptoms than with depression symptoms in the three samples of patients (*z* = 3.5, *p* = .001; *z* = 4.2, *p* ≤ .001; *z* = 3.3, *p* ≤ .001; for GAD, MDD and MADD, respectively). In turn, brooding was more strongly associated with depressive symptoms than with anxiety symptoms only in the MDD group (*z* = 2.0, *p* = 0.05), whereas there were no significant differences in the magnitude of the correlations involving reflection in any of the cases.

**Table 2:** Correlations among Neuroticism, Worry, Brooding, Reflection and Emotional Symptoms by Diagnostic Group. GAD, Generalized Anxiety Disorder; MDD, Major Depressive Disorder; MADD, Mixed Anxiety-Depressive Disorder.

Variables	1	2	3	4	5
**GAD (*n* = 45)**					
1. Neuroticism	__				
2. Worry	.62***	__			
3. Brooding	.53***	.48**	__		
4. Reflection	.13	.32*	.51**	__	
5. Anxiety symptoms	.73***	.80***	.49**	.15	__
6. Depressive symptoms	.68***	.55***	.65***	.08	74***
**MDD (*n* = 49)**					
1. Neuroticism	__				
2. Worry	.48**	__			
3. Brooding	.71***	.30*	__		
4. Reflection	.15	.20	.20	__	
5. Anxiety symptoms	.61***	.74***	.53**	.26	__
6. Depressive symptoms	.61***	.23	.74***	.26	.42**
**MADD (*n* = 40)**					
1. Neuroticism	__				
2. Worry	.58***	__			
3. Brooding	.64***	.58***	__		
4. Reflection	.20	.04	.38*	__	
5. Anxiety symptoms	.68***	.76***	.65***	-.02	__
6. Depressive symptoms	.65***	.42**	.75***	.31	.62***

*p < .05.

**p < .01.

***p < .001.

### Multiple mediation analyses

Multiple mediation analyses were conducted using the INDIRECT macro for SPSS [44]. As the starting point of mediation was the existence of a predictive effect of neuroticism on anxious/depressive symptoms, the first step was to test the univariate relationship between these variables in linear regression analyses for each diagnostic group [45], considering neuroticism as the independent variable and anxiety or depressive symptoms as the dependent variables. Gender and depressive or anxious symptoms, respectively, were controlled for in these analyses. The results indicated the existence of a predictive effect of neuroticism on both anxious and depressive symptoms in all diagnostic groups (*β* ranged from 1.57 to 2.61, *p* ≤ .05), except in the case of the GAD group for depressive symptoms, although with a probability close to statistical significance (*β* = 1.08, *p* = .06).

Multiple mediation analyses were then conducted. Bias-corrected bootstrapping (with 20 000 resamples) was used to generate confidence intervals for the hypotheses tested because it is the preferred method for assessing indirect effects in both simple [46] and multiple mediator models [44]. In addition, as bootstrapping involves repeatedly sampling from the data set and estimating the indirect effect in each resampled date set, it is appropriate for small to moderate sample. For each diagnostic group, two multiple mediator models were tested in which neuroticism was the independent variable, the cognitive variables (worry, brooding and reflection) were the potential mediators and anxiety or depressive symptoms were the dependent variables, respectively. Anxiety symptoms were controlled for in the analyses where depressive symptoms were the dependent variable, and vice versa. Gender was controlled for across all analyses as well. A multiple mediation strategy was used because it allows estimation of both the total indirect effect associated with all mediators and the specific indirect effect associated with each mediator. Pairwise contrasts of all the specific indirect effects involved in each model were also calculated.

The results for the GAD group are summarized in Table 3. Regarding the analysis predicting anxiety symptoms, the total indirect effect of the three proposed mediators was statistically significant (i.e., the bias-corrected 95% confidence interval for this effect did not contain zero). Although not shown in the table, the joint effect of worry, brooding and reflection reduced the non-standardized regression coefficient of neuroticism on anxiety symptoms from 1.57 (*p* ≤ .01) to 0.85 (*p* = .06), thus accounting for 45.86% [(1.57–0.85)/1.57] of the association between neuroticism and anxiety symptoms and rendering the effect of neuroticism non-significant (which is usually referred as “full mediation” [45]). When examining the specific indirect effect of each cognitive variable, only worry emerged as a significant mediator of the relationship between neuroticism and anxiety symptoms. Furthermore, the pairwise contrasts revealed that the specific indirect effect of neuroticism on anxiety symptoms through worry was significantly larger than those through brooding and reflection. As for the analysis predicting depressive symptoms in this diagnostic group, neither the total indirect effect of the three cognitive variables nor the specific indirect effect of any of them reached statistical significance, although the specific indirect effect of neuroticism on depressive symptoms through brooding was near-significant. The pairwise contrasts of the specific indirect effects did not yield any significant result either, so that there were no significant differences in the mediating power of worry, brooding and reflection.

**Table 3:** Results of Multiple Mediation Analyses for the GAD Group. GAD, Generalized Anxiety Disorder; BC 95% CI, bias-corrected 95% confidence interval.

Mediation Pathway	Point Estimate	*SE*	BC 95% CI	
			Lower	Upper
**Neuroticism → Cognitive variables → Anxiety symptoms**				
*Indirect effects*				
TOTAL	0.72	0.39	0.12	1.74
Worry	0.79	0.39	0.21	1.85
Brooding	-0.06	0.14	-0.64	0.08
Reflection	-0.01	0.11	-0.37	0.14
*Contrasts*				
Worry vs. brooding	0.85	0.42	0.19	1.93
Worry vs. reflection	0.81	0.41	0.19	1.92
Brooding vs. reflection	-0.05	0.21	-0.64	0.28
**Neuroticism → Cognitive variables → Depressive symptoms**				
*Indirect effects*				
TOTAL	0.55	0.45	-0.05	1.86
Worry	-0.04	0.11	-0.42	0.08
Brooding	0.64	0.45	-0.01	1.85
Reflection	-0.05	0.23	-0.68	0.31
*Contrasts*				
Worry vs. brooding	-0.68	0.47	-1.88	0.04
Worry vs. reflection	0.01	0.25	-0.46	0.59
Brooding vs. reflection	0.69	0.55	-0.14	2.14

The results for the MDD group are presented in Table 4. With respect to the analysis predicting anxiety symptoms, the total indirect effect of the three proposed mediators was statistically significant and reduced the non-standardized regression coefficient of neuroticism on anxiety symptoms from 2.08 (*p* ≤ .001) to 0.47 (*p* = .35), which entails a full mediation effect explaining 77.40% [(2.08–0.47)/2.08] of the association between both variables. When exploring the specific indirect effect of each cognitive variable, only worry turned out to be a significant mediator of the relationship between neuroticism and anxiety symptoms. The pairwise contrasts showed that the specific indirect effect of neuroticism on anxiety symptoms through worry was significantly larger than that through reflection; however, the specific indirect effects through worry and brooding were not distinguished from each other in terms of magnitude, despite the fact that the former was significantly different from zero and the latter was not (such apparent paradoxes can occur when one of the specific indirect effects included in the contrast is not sufficiently far from zero [44]). As regards the analysis aimed at predicting depressive symptoms, the total indirect effect of the three proposed mediators was found to be significant and involved a reduction in the non-standardized regression coefficient of neuroticism on depressive symptoms from 2.61 (*p* ≤ .001) to 1.00 (*p* = .17), thereby being a full mediation effect responsible for 61.68% [(2.61–1.00)/2.61] of the association between both variables. Among the specific indirect effects within this analysis, only that through brooding was statistically significant. Moreover, the pairwise contrasts revealed that the specific indirect effect of neuroticism on depressive symptoms through brooding was significantly greater in magnitude than those through worry and reflection.

**Table 4:** Results of Multiple Mediation Analyses for the MDD Group. MDD, Major Depressive Disorder; BC 95% CI, bias-corrected 95% confidence interval.

Mediation Pathway	Point Estimate	*SE*	BC 95% CI	
			Lower	Upper
**Neuroticism → Cognitive variables → Anxiety symptoms**				
*Indirect effects*				
TOTAL	1.61	0.52	0.71	2.81
Worry	1.25	0.41	0.46	2.10
Brooding	0.37	0.27	-0.08	1.03
Reflection	-0.01	0.07	-0.19	0.14
*Contrasts*				
Worry vs. brooding	0.88	0.49	-0.08	1.87
Worry vs. reflection	1.25	0.41	0.46	2.10
Brooding vs. reflection	0.37	0.27	-0.09	1.00
**Neuroticism → Cognitive variables → Depressive symptoms**				
*Indirect effects*				
TOTAL	1.61	0.60	0.59	3.04
Worry	-0.01	0.13	-0.45	0.14
Brooding	1.63	0.53	0.77	2.96
Reflection	-0.01	0.15	-0.37	0.28
*Contrasts*				
Worry vs. brooding	-1.65	0.54	-2.97	-0.77
Worry vs. reflection	-0.01	0.19	-0.44	0.36
Brooding vs. reflection	1.64	0.53	0.77	2.96

Finally, the results obtained for the MADD group can be seen in Table 5. Respecting the prediction of anxiety symptoms, the total indirect effect of the three proposed mediators was statistically significant and reduced the non-standardized regression coefficient of neuroticism on anxiety symptoms from 2.20 (*p* ≤ .01) to 0.69 (*p* = .27), which implies a full mediation effect capable of explaining 68.64% [(2.20–0.69)/2.20] of the association between both variables. When analyzing the specific indirect effect of each cognitive variable, only worry emerged as a significant mediator between neuroticism and anxiety symptoms in this diagnostic group. In addition, the pairwise contrasts indicated that the specific indirect effect of neuroticism on anxiety symptoms through worry was significant larger than those specific indirect effects involving both brooding and reflection. Regarding the prediction of depressive symptoms, the total indirect effect carried by the three proposed mediators was statistically significant and reduced the non-standardized regression coefficient of neuroticism on depressive symptoms from 1.94 (*p* ≤ .05) to 1.13 (*p* = .10), thereby emerging as a full mediation effect accounting for 41.75% [(1.94–1.13)/1.94] of the association between both variables. Concerning the individual contribution of each mediating pathway, only the specific indirect effect of neuroticism on depressive through brooding reached statistical significance. Pairwise contrasts also revealed that the mediating effect of brooding was larger than that of worry, although there were no significant differences in the magnitude of the mediating effects of brooding and reflection.

**Table 5:** Results of Multiple Mediation Analyses for the MADD Group. MADD, Mixed Anxiety-Depressive Disorder; BC 95% CI, bias-corrected 95% confidence interval.

Mediation Pathway	Point Estimate	*SE*	BC 95% CI	
			Lower	Upper
**Neuroticism → Cognitive variables → Anxiety symptoms**				
*Indirect effects*				
TOTAL	1.50	0.54	0.50	2.62
Worry	1.21	0.49	0.43	2.36
Brooding	0.20	0.26	-0.13	0.95
Reflection	0.10	0.25	-0.31	0.73
*Contrasts*				
Worry vs. brooding	1.01	0.60	0.01	2.39
Worry vs. reflection	1.11	0.54	0.16	2.30
Brooding vs. reflection	0.10	0.40	-0.53	1.10
**Neuroticism → Cognitive variables → Depressive symptoms**				
*Indirect effects*				
TOTAL	0.81	0.53	0.03	2.17
Worry	-0.16	0.20	-0.73	0.11
Brooding	0.74	0.43	0.13	1.90
Reflection	0.23	0.26	-0.03	1.15
*Contrasts*				
Worry vs. brooding	-0.90	0.50	-2.14	-0.12
Worry vs. reflection	-0.39	0.33	-1.37	0.04
Brooding vs. reflection	0.50	0.49	-0.34	1.63

## Discussion

The current study sought to investigate the relationships between neuroticism (higher-order vulnerability factor), the cognitive styles of worry, brooding and reflection (second-order vulnerability factors) and symptoms of anxiety and depression in three groups of patients (GAD, MDD and MADD).

As a preliminary step, we found that the cognitive processes worry and brooding-rumination tend to be significantly associated with anxiety and depressive symptoms in all the groups. However, worry is not significantly associated with depressive symptoms in the MDD group. As previously suggested [20], this may reflect the role of brooding as the preferred cognitive strategy underlying depressive symptoms in pure depressed patients, perhaps because the over-elaborate processing of the emotional state inherent in depressive rumination is particularly relevant in these patients, whereas worry about future threats may not make a real contribution to the development of depressive symptoms once depressive affect is clinically present and has become the main emotion. This also appears to indicate that worry and brooding are distinct cognitive processes that play a differential role in GAD and MDD, as shown in another study with a bigger clinical sample where worry and brooding proved useful in distinguishing between GAD and MDD at the cognitive level [47]. On the other hand, reflection-rumination is not associated with psychopathology in any group. Thus, it seems that, although reflection shares the same repetitive thought processes as brooding and worry, it may differ in valence, the context in which it occurs and the level of construal adopted [21]. It is possible that the purposeful turning inward to engage in cognitive problem solving to alleviate depressive symptoms is a less maladaptive form of rumination [18], although more research is warranted to further elucidate the precise role of reflection in emotional symptomatology.

According to the results in relation to the main aim of the study, the total indirect effects observed suggest that the proposed cognitive variables (worry, brooding and reflection) as a whole mediate, on the one hand, the relationship between neuroticism and anxiety symptoms in the three groups of patients and, on the other hand, the relationship between neuroticism and depressive symptoms in the groups of patients diagnosed with MDD or MADD. It is noteworthy that the significant total indirect effects found were large in magnitude, explaining between 41.75% and 77.40% of the association between neuroticism and emotional symptoms and rendering the direct effect of neuroticism non-significant, which supports full mediation. These results might be interpreted in terms that the relation between neuroticism and emotional symptoms is really spurious, as it disappears once the cognitive variables are incorporated into the analyses (confounding effect). However, the literature documents the role of neuroticism as an undifferentiated disposition that truly increases the risk of developing anxious and depressive symptoms through intervening cognitive variables that act as an intermediate step in the vulnerability chain [5,48,49]. Given this evidence and following the conceptual distinction between mediation and confounding effects [50], we think that our findings are in accordance which a hierarchical model of vulnerability in which neuroticism is a distal risk factor leading to more proximal risk factors involving repetitive thinking processes, which in turn lead to emotional symptoms. In any case, these results need to be replicated in prospective studies.

Beyond this general information, specific routes of mediation are observed when focusing on the relative contribution of each mediator. In particular, worry always acts as a significant mediating mechanism between neuroticism and anxiety symptoms, not only in patients in whom anxiety is the principal emotion (GAD), but also in patients whose principal emotion is depression (MDD) and in those in whom symptoms of anxiety and depression coexist without any clear predominance of one over the other (MADD). The pairwise contrasts also show that worry is clearly a better mediator between neuroticism and anxiety symptoms than brooding in those diagnoses involving anxiety (GAD and MADD), but not in MDD and, therefore, brooding-rumination might have a marginal mediating effect on anxiety symptoms in “pure” depressed patients. With respect to brooding, it significantly mediates the relationship between neuroticism and symptoms of depression in patients with MDD and MADD, but not in patients with GAD, although considering the confidence interval found for the indirect effect of brooding in these patients it cannot be excluded that such a mediating effect exists. The pairwise contrasts also reveal that the indirect effect of brooding on depressive symptoms is significantly larger than those of worry and reflection in MDD, larger than that of worry in MADD and not larger than any other indirect effect in GAD, which suggests that the mediating effect of brooding is increasingly specific as depression is more central to the patient’s diagnosis. Finally, it should also be noted that reflection-rumination does not appear to play any significant mediating role in any case, in accordance with previous results [18,51,52].

Taking the above into account, as worry appears to be the preferred mechanism whereby neuroticism is linked to anxiety symptoms and brooding-rumination appears to be the main pathway via which neuroticism carries its effect to depressive symptoms, the results of the mediation analyses are consistent with previous findings supporting specificity [47,53,54]. It should be noted, however, that although the cognitive processes of worry and brooding-rumination tend to be specific at the symptom level (anxious vs. depressive symptoms), the mediation results are quite similar in the three groups of patients and, consequently, are not exclusive to a disorder. In this regard, specificity should be considered as a relative phenomenon rather than as an absolute one [3]. Indeed, it is interesting to emphasize that the specificity by which, from high levels of neuroticism, worry leads to anxious symptoms and brooding-rumination leads to depressive symptoms not only occurs in pure anxious or depressed patients, respectively, but also in patients undergoing mixed anxious-depressive symptoms in whom the studied constructs may be thought to be less predictable or intrinsically associated with the diagnosis. As a result, we may posit that worry and brooding-rumination appear to be specific pathways toward the development of anxious or depressive symptoms, respectively, but acting as transdiagnostic processes common to different emotional disorders. This would fit with what could be referred to as a “transdiagnostic specificity” hypothesis.

## Conclusions

This study tested the transdiagnostic and content specificity hypotheses. Overall, our findings indicate that both approaches may be combined resulting in "specific transdiagnostic” mechanisms. On the one hand, the results support specificity insomuch as the mediation analyses confirm the worry-anxious symptoms and brooding-depressive symptoms links suggested in the literature. In line with this, although worry and brooding share characteristics such as excessive and repetitive thinking [21], they differ in relation to content, temporal orientation, controllability and certainty of the stressful events on which they focus, and they are differently associated with the symptoms in relation to the anxiety-mood dichotomy [53,55,56]. On the other hand, the transdiagnostic hypothesis is also corroborated to the extent that worry and brooding both appear to operate in the three diagnostic groups. Although the cross-sectional nature of the present study prevents us from establishing the direction of the relationships, the findings also dovetail with a hierarchical model of vulnerability in which the cognitive reactivity of individuals who score highly on neuroticism (i.e., they tend to engage in repetitive and uncontrollable thought processes) may increase the risk of anxious and depressive symptoms via selective links involving either worry or brooding, respectively.

## Limitations and Clinical Implications

The following limitations of the study must be addressed. First, the cross-sectional design of the study precludes conclusions being made about the direction of the effects. Although the majority of research suggests the directionality modelled in the present study (neuroticism predicting cognitive variables and cognitive variables predicting anxiety and depressive symptoms), reverse effects cannot be ruled out [57,58,59]. Second, the sample was relatively small, which not only limits the general applicability of our findings, but also diminishes the statistical power of the study given the number of parameters estimates [60]. Third, only self-report measures were used, which may have introduced some bias in the results. Finally, multicollinearity may have played a role in our results, which seems to be intrinsically associated with multiple mediation models because the mediators tend to be correlated by virtue of their mutual reliance on a common cause [44]. In this regard, albeit multicollinearity does not compromise the reliability of the model, it should be acknowledged that the specific indirect effects may have been attenuated due to content overlap between the mediators.

In terms of potential clinical implications, the identification of cognitive mechanisms that specifically link neuroticism and emotional psychopathology underlines the importance of adapting therapeutic interventions, taking into account the particular characteristics of worriers (uncertain and potentially controllable events) and ruminators (certain and uncontrollable events). Provided our findings were replicated in prospective studies, programs aimed at reducing worry might help patients with GAD, MDD or MADD decrease their level of anxiety symptoms, whereas treatment strategies targeting brooding-rumination could be effective in reducing depressive symptoms in patients with emotional disorders, particularly those whose depression is more salient. In this regard, there appear to have emerged innovative and promising interventions addressing either worry or rumination, such as metacognitive therapy [61], which focuses on the beliefs of the patient about worry, rather than the process itself, or cognitive-behavioural therapy centred on rumination [62], which enables patients to develop new strategies aimed at meeting challenges and is less marked by a self-referenced perspective directed towards engaging in rumination.
